# Maternal predictors of early-onset sepsis in neonates: a multicenter retrospective cohort study and risk prediction model

**DOI:** 10.1186/s12967-025-07154-2

**Published:** 2025-10-16

**Authors:** Shu Li, Yuqing Jiang, Hongmei Hu, Jialin Qu, Dachuan Zeng, Yuping Ran, Zhu Luo, Lihua Kang, Jianfeng Gong, Yingying Wu, Kewei Xing, Chunli Li

**Affiliations:** 1 Department of Clinical Laboratory, Women and Children’s Hospital of Chongqing Medical University, Chongqing, 401174 China; 2 Department of Clinical Laboratory, Chongqing Health Center for Women and Children, 120 Longshan Road, Chongqing, 401174 China; 3 NHC Key Laboratory of Birth Defects and Reproductive Health, Chongqing, 401174 China; 4 Chongqing Research Center for Prevention and Control of Maternal and Child Diseases and Public Health, Chongqing, 401174 China; 5 Department of Clinical Laboratory, Yunyang Maternal and Child Healthcare Hospital, Chongqing, 404500 China

**Keywords:** Early-onset sepsis, Maternal health, Neonates, Laboratory indicators

## Abstract

**Objectives:**

Early-onset sepsis (EOS) is a common and serious neonatal infection that contributes significantly to morbidity and mortality. A comprehensive understanding of maternal risk factors and their association with EOS is essential for developing effective preventive strategies.

**Methods:**

In this study, Clinical and laboratory data of the patients were extracted from two hospitals in Chongqing, China (2017–2023). Collinearity analysis was performed to exclude variables with multicollinearity. Restricted cubic spline (RCS) analyses were applied to evaluate potential linear or nonlinear associations between continuous maternal variables and the risk of EOS. A multivariable Poisson regression model was developed to identify independent maternal predictors of EOS and construct a predictive model. Model performance was assessed using the area under the receiver operating characteristic curve (AUC). In addition, a nomogram based on the final Poisson regression model was established to enable individualized risk prediction and support clinical decision-making.

**Results:**

This multicenter retrospective cohort study included 79,570 mother-infant pairs, the incidence of EOS was 0.56%. Univariate and multivariable Poisson regression identified key risk factors, such as chorioamnionitis, puerperal infection, intrauterine fetal distress, preterm birth, twin pregnancy, and elevated maternal WBC, HGI, M%, and PWR. Conversely, higher maternal globulin and cesarean delivery were associated with reduced EOS risk. A predictive model with good discrimination (AUC = 0.762) was developed and visualized through a nomogram to facilitate individualized risk assessment.

**Conclusion:**

This study highlights the importance of maternal factors in early EOS prediction and supports their integration into perinatal risk assessment. Further research is needed to investigate the contribution of maternal psychosocial and nutritional status and validate the model across diverse populations.

**Supplementary Information:**

The online version contains supplementary material available at 10.1186/s12967-025-07154-2.

## Introduction

Neonatal sepsis remains one of the most critical threats to newborn survival [1]. Based on the timing of disease onset, neonatal sepsis is typically classified into early-onset sepsis (EOS), occurring within the first 72 h of life, and late-onset sepsis (LOS) [1]. EOS is often characterized by nonspecific symptoms and subtle clinical signs, which hinder timely diagnosis and increases the risk of adverse outcomes [2].

EOS is usually caused by vertical transmission of pathogens from the mother to the neonate during the perinatal period [3]. The main causative agents are bacteria, particularly Group B Streptococcus and Gram-negative organisms such as *Escherichia coli* [2, 4–6]. EOS typically presents with an acute onset and is frequently accompanied by pneumonia and multi-organ involvement [5]. EOS is associated with high mortality, making early diagnosis and timely treatment essential for improving neonatal survival [7]. Given the substantial physiological and clinical differences between neonates and older children, the diagnostic criteria for neonatal sepsis have been continuously evolving, along with the predictive indicators [8]. Currently, clinical assessment of neonatal inflammatory status primarily relies on biomarkers such as white blood cell (WBC) count and the proportion of neutrophils [9]. While these markers reflect the degree of inflammation and disease progression, they mainly capture existing inflammation and are insufficient for predicting the onset of neonatal sepsis. In clinical practice, the prediction of EOS requires a comprehensive assessment of multiple factors, including clinical manifestations, laboratory findings, maternal-related variables, and neonatal-related variables, enabling timely evaluation and intervention [10]. To minimize invasive procedures in newborns and facilitate early identification of EOS risk, this study investigated maternal risk factors associated with the occurrence of EOS and developed a predictive model.

## Methods and materials

### Patients

This multicenter retrospective cohort study collected clinical data of neonates admitted to the neonatal departments and their mothers from January 2017 to December 2023 at Chongqing Health Center for Women and Children and Yunyang Maternal and Child Healthcare Hospital. The study protocol was approved by the Ethics Committee of Chongqing Health Center for Women and Children (Approval No.: 2023 ethics department 019) and conducted in accordance with the ethical principles of the Declaration of Helsinki. As this was an anonymized retrospective study, the requirement for informed consent was waived by the ethics committee.

### Inclusion and exclusion criteria

Inclusion criteria: (1) Neonates admitted within ≤ 72 h after birth; (2) availability of complete maternal pregnancy and delivery records; (3) neonates with a confirmed diagnosis of EOS, or enrolled as controls (no signs of infection and no diagnosis of infection during hospitalization). The diagnosis of EOS was based on the Expert Consensus on the Diagnosis and Treatment of Neonatal Sepsis [11].

Exclusion criteria: (1) Neonates with congenital malformations or chromosomal abnormalities; (2) Missing key clinical or laboratory data; (3) Neonatal infections with a confirmed nonmaternal (vertical) source, such as healthcare-associated infections; (4) Neonates born from terminated pregnancies or those who were not live births.

### Data collection

Clinical and laboratory data were extracted from the hospital’s electronic medical record system (Hospital Information System, HIS) and laboratory information system (LIS). Neonatal data included sex, multiple birth status, preterm birth, macrosomia, and signs of neonatal distress, among others. Maternal data included pregnancy-related medical history (such as gestational diabetes mellitus, gestational hypertension, and intrahepatic cholestasis of pregnancy), mode of delivery, and antenatal infections, etc. Maternal laboratory indicators were obtained from the first tests performed upon admission and included complete blood count (e.g., white blood cell count, hemoglobin, platelets), liver function tests (e.g., globulin, total bilirubin), and renal function tests (e.g., creatinine, UREA). All data were independently extracted and verified by two trained researchers to ensure completeness and accuracy.

### Calculation of derived indices

Several commonly used derived hematological and biochemical indices were calculated based on the following formulas: platelet-to-white cell ratio (PWR) = platelet (PLT)/WBC; neutrophil-to-lymphocyte ratio (NLR) = neutrophil absolute count (N#)/lymphocyte absolute count (L#); monocyte-to-lymphocyte ratio (MLR) = monocyte absolute count (M#)/L#; neutrophil-monocyte-to-lymphocyte ratio (NMLR) = (M# + N#)/L#; systemic inflammation response index (SIRI) = (N# × M#)/L#; systemic immune-inflammation index (SII) = (PLT × N#)/L#; derived neutrophil-to-lymphocyte ratio (DNLR) = N#/(WBC − L#); aspartate aminotransferase (AST) to alanine aminotransferase (ALT) Ratio (SLR) = AST/ALT; UREA-to-creatinine ratio (UCR) = UREA/creatinine (CR); hemoglobin glycemic index (HGI) = HbA1c − Pre_HbA1c; prognostic nutritional index (PNI) = 10 × albumin (ALB) + 0.005 × L#. These indices were used to evaluate maternal inflammatory and metabolic status in relation to the risk of neonatal EOS.

### Statistical analysis

All statistical analyses were performed using R software (version 4.5.0). Normality of continuous variables was assessed using the Shapiro-Wilk test. The normality of continuous variables was assessed using the Shapiro-Wilk test. Normally distributed data were expressed as mean ± standard deviation (Mean ± SD) and compared using the independent t-test, whereas non-normally distributed data were presented as median with interquartile range (IQR) and compared using the Mann–Whitney U test. Categorical variables were presented as counts and percentages, and group comparisons were conducted using the Chi-square test or Fisher’s exact test, as appropriate.

Univariate analyses were conducted to identify maternal risk factors associated with EOS. For categorical variables, Chi-square tests were performed to evaluate the association between each variable and the risk of EOS. For continuous variables, two approaches were employed to ensure robustness in variable selection. First, cut-off values were determined for each variable, which were then used to dichotomize the continuous variables, followed by Chi-square tests. Second, restricted cubic spline analyses were performed to explore potential linear or nonlinear associations between continuous variables and EOS. Variables identified as significant by either approach were visualized using Venn diagrams, and overlapping variables were selected as candidates for the multivariate model to improve stability and interpretability.

Prior to multivariate regression, multicollinearity among independent variables was assessed using the variance inflation factor (VIF) and tolerance. A VIF > 10 or a tolerance < 0.1 was considered indicative of severe multicollinearity. Variables exhibiting multicollinearity were reduced or combined based on clinical relevance and statistical principles to optimize model stability. Variables identified as significant in the univariate analysis were entered into a multivariate Poisson regression model to determine independent risk factors, with incidence rate ratio (IRR) and 95% confidence intervals (CIs) calculated. Model performance was evaluated using the area under the receiver operating characteristic curve. We developed an initial model (Model 1) and a final model (Model 2). To assess the stability of the final EOS prediction model and potential overfitting, we first constructed the model using logistic regression to verify consistency with Poisson regression results. Model 2 also was subjected to 1000 bootstrap resamples. For each bootstrap sample, the model was refitted, and the AUC, Brier score, as well as the calibration intercept and slope were calculated. These metrics were then applied to the original dataset to estimate model optimism. In addition, a nomogram based on the final multivariate Poisson regression model was constructed to facilitate individualized risk prediction and clinical application. R (version 4.5.0) and Origin 2024 software were used for data visualization. All statistical tests were two-sided, and a *P*-value < 0.05 was considered statistically significant.

## Results

### Basic information

**Fig. 1:**
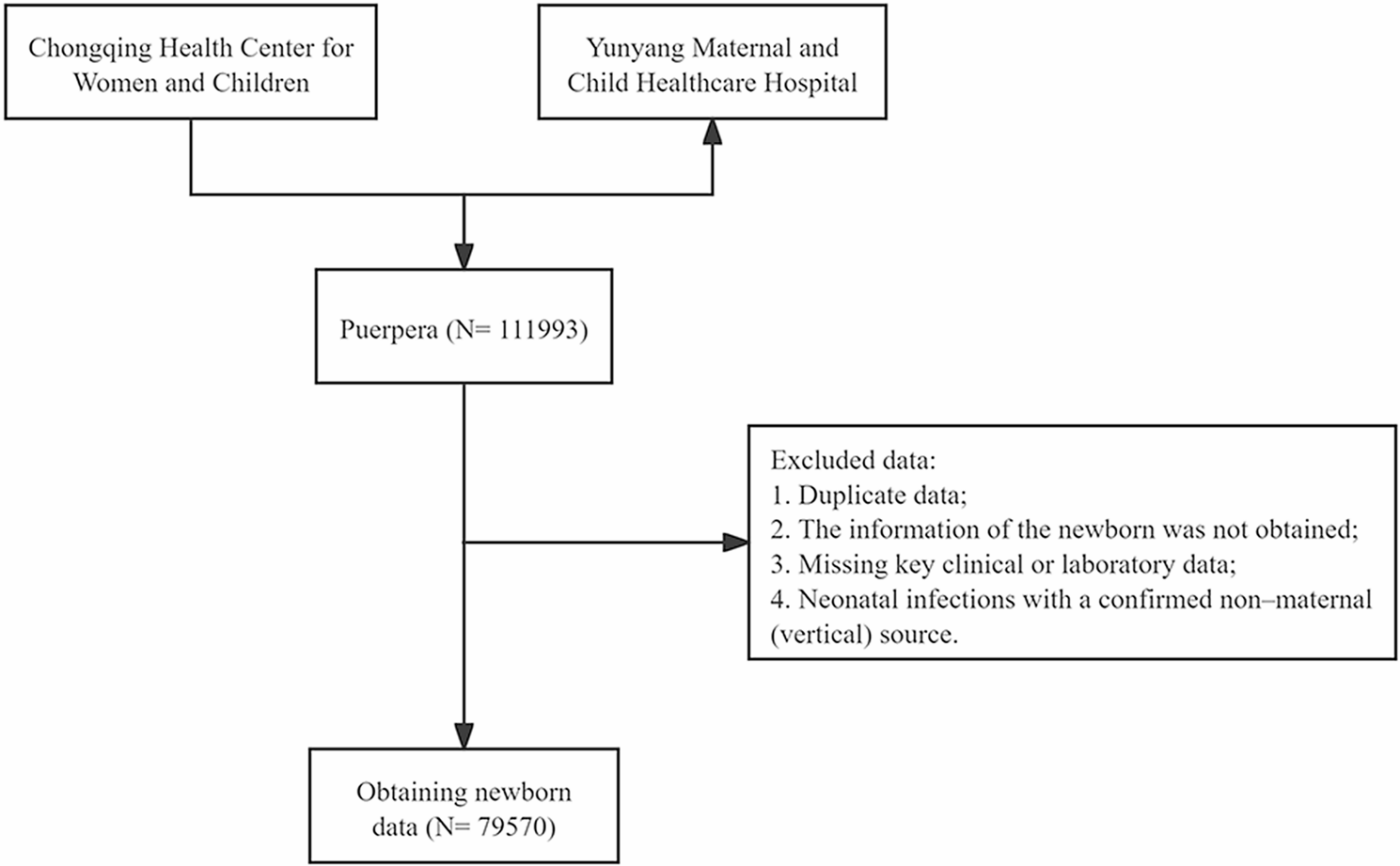
Flowchart of patient selection

From 2017 to 2024, a total of 111,993 pregnant women were included in this study, of whom 79,570 were successfully matched with corresponding neonatal records (Fig. 1). The overall incidence of EOS was 0.56% (449/79,570). No statistically significant differences were observed between the EOS-positive and EOS-negative groups with respect to maternal age, pre-pregnancy weight, height, pre-pregnancy body mass index (BMI), premature rupture of membranes (PROM), threatened preterm labor (TPL), fetal growth restriction (FGR), abnormal obstetric history (AOH), smoking history, or alcohol consumption history (ACH) (Supplementary Table 1). Compared with the control group, mothers of neonates who developed EOS exhibited significantly lower levels of ALB, PNI, PA, MCV, A/G ratio, and SLR, whereas levels of MLR, SII, GGT, NMLR, and NLR were markedly elevated; all differences were statistically significant (all *P* < 0.05) (Table 1). To further explore the association between maternal age and EOS risk, maternal age was categorized into five groups: < 25, 25–29, 30–34, 35–39, and ≥ 40 years. The EOS incidence was highest in the 25–29 years (0.60%) and 30–34 years (0.61%) age groups, and the difference was statistically significant (*P* < 0.001) (Fig. 4a).

**Table 1:** Basic parameters of continuous variables

Variables	Control group (Mean ± SD)	Control group, *N*	EOS (Mean ± SD)	EOS, *N*	*P*
ALB	34.77 ± 2.78	79,141	34.1 ± 3.2	429	< 0.001
UCR	0.08 ± 0.02	79,141	0.08 ± 0.02	429	< 0.001
PNI	347.67 ± 27.84	79,141	341.06 ± 32.02	429	< 0.001
PA	210.79 ± 34.62	79,141	201.79 ± 40.21	429	< 0.001
MCV	91.77 ± 6.1	79,141	90.65 ± 6.18	429	< 0.001
RBC	3.89 ± 0.45	79,141	3.96 ± 0.47	429	< 0.001
A/G	1.18 ± 0.38	79,141	1.16 ± 0.3	429	0.002
L#	1.55 ± 0.48	79,141	1.47 ± 0.46	429	0.002
MLR	0.38 ± 0.18	79,141	0.43 ± 0.28	429	0.002
UREA	3.72 ± 1.96	79,141	3.65 ± 2.7	429	0.002
SII	1041.86 ± 696.97	79,141	1213.21 ± 974.6	429	0.003
MCH	31.04 ± 2.58	79,141	30.73 ± 2.6	429	0.008
Pre-pregnancy weight	54.26 ± 8.79	71,268	55.06 ± 8.13	376	0.011
Neonatal weight	3231.08 ± 491.16	67,677	3024.21 ± 794.78	385	0.013
GGT	16.47 ± 16.5	79,141	18.01 ± 16.36	429	0.023
L%	16.05 ± 5.56	79,141	15.32 ± 6.21	429	0.024
NMLR	6.21 ± 3.77	79,141	7.29 ± 5.68	429	0.026
SLR	1.5 ± 0.66	79,141	1.46 ± 0.65	429	0.033
NLR	5.83 ± 3.63	79,141	6.86 ± 5.44	429	0.033
TP	64.85 ± 4.86	79,141	64.27 ± 5.81	429	0.033
IBIL	7.38 ± 2.6	79,141	7.22 ± 2.99	429	0.033
HGB	120.09 ± 13.55	79,141	121.03 ± 14.45	429	0.038
ALP	159.53 ± 64.15	79,141	155.47 ± 62.04	429	0.045
SIRI	3.36 ± 3.11	79,141	4.47 ± 6	429	0.051
M#	0.55 ± 0.19	79,141	0.57 ± 0.22	429	0.054
RDWSD	46.13 ± 4.02	79,141	45.76 ± 3.93	429	0.070
LDH	177.2 ± 39.15	79,141	182.45 ± 48.61	429	0.071
M%	5.42 ± 1.36	79,141	5.5 ± 1.49	429	0.076
HCT	35.51 ± 3.63	79,141	35.71 ± 3.9	429	0.080
Pre-pregnancy BMI	21.44 ± 3.26	64,093	21.63 ± 2.89	324	0.091
N%	77.68 ± 6.4	79,141	78.33 ± 7.23	429	0.095
TBIL	9.22 ± 3.32	79,141	9.14 ± 3.76	429	0.115
CYSC	1.27 ± 0.3	79,141	1.29 ± 0.32	429	0.145
RDW	13.91 ± 1.26	79,141	14.02 ± 1.36	429	0.147
ALT	18.23 ± 29.39	79,141	24.14 ± 51.86	429	0.161
MCHC	337.93 ± 11.26	79,141	338.67 ± 11.69	429	0.203
Age	30.02 ± 4.02	79,141	29.76 ± 3.59	429	0.261
CR	46.95 ± 14.27	79,141	47.59 ± 16.77	429	0.284
Height	1.59 ± 0.05	64,456	1.59 ± 0.05	329	0.291
TBA	4.53 ± 5.62	79,141	5.26 ± 8.27	429	0.409
PLT	180.3 ± 51.57	79,141	183.11 ± 54.68	429	0.433
E#	0.06 ± 0.06	79,141	0.06 ± 0.08	429	0.452
B#	0.23 ± 0.13	79,141	0.23 ± 0.13	429	0.482
DNLR	0.92 ± 0.02	79,141	0.92 ± 0.03	429	0.487
E%	0.62 ± 0.67	79,141	0.61 ± 0.72	429	0.508
DBIL	1.86 ± 1.34	79,141	1.94 ± 1.33	429	0.519
N#	8.15 ± 3.12	79,141	8.61 ± 4.05	429	0.608
AST	21.31 ± 19.02	79,141	24.26 ± 27.93	429	0.610
WBC	10.33 ± 3.31	79,141	10.74 ± 4.14	429	0.661
UA	331.56 ± 82.05	79,141	334.16 ± 89.4	429	0.817
PWR	18.97 ± 7.42	79,141	19.22 ± 8.5	429	0.896
GLB	30.07 ± 3.61	79,141	30.16 ± 4.06	429	0.908
B#	0.02 ± 0.01	79,141	0.02 ± 0.01	429	0.933

EOS, early-onset sepsis; SD, standard deviation; N, number of patients; WBC, white blood cell count; PLT, platelet; RBC, red blood cell count; HGB, hemoglobin; HCT, hematocrit; MCV, mean corpuscular volume; MCH, mean corpuscular hemoglobin; MCHC, mean corpuscular hemoglobin concentration; RDW, red cell distribution width; RDWSD, red cell distribution width (standard deviation); N%, neutrophil percentage; M%, monocyte percentage; N#, neutrophil absolute count; M#, monocyte absolute count; L%, lymphocyte percentage; E%, eosinophil percentage; L#, lymphocyte absolute count; E#, eosinophil absolute count; B%, basophil percentage; B#, basophil absolute count; PA, prealbumin; TP, total protein; ALB, albumin; GLB, globulin; A/G, albumin/globulin ratio; TBIL, total bilirubin; DBIL, direct bilirubin; IBIL, indirect bilirubin; TBA, total bile acids; ALT, alanine aminotransferase; AST, aspartate aminotransferase; ALP, alkaline phosphatase; GGT, gamma-glutamyl transferase; LDH, Lactate dehydrogenase; CR, creatinine; UA, uric acid; CYSC, cystatin C; PWR, platelet to white cell ratio; NLR, neutrophil to lymphocyte ratio; MLR, monocyte to lymphocyte ratio; NMLR, (neutrophil + monocyte) to lymphocyte ratio; SIRI, systemic inflammation response index; SII, systemic immune-inflammation index; DNLR, derived neutrophil to lymphocyte ratio; SLR, AST to ALT ratio; UCR, UREA to creatinine ratio; HGI, hemoglobin to glycerinated hemoglobin index; PNI, prognostic nutrition index

### The maternal categorical factors associated with the risk of EOS

**Fig. 2:**
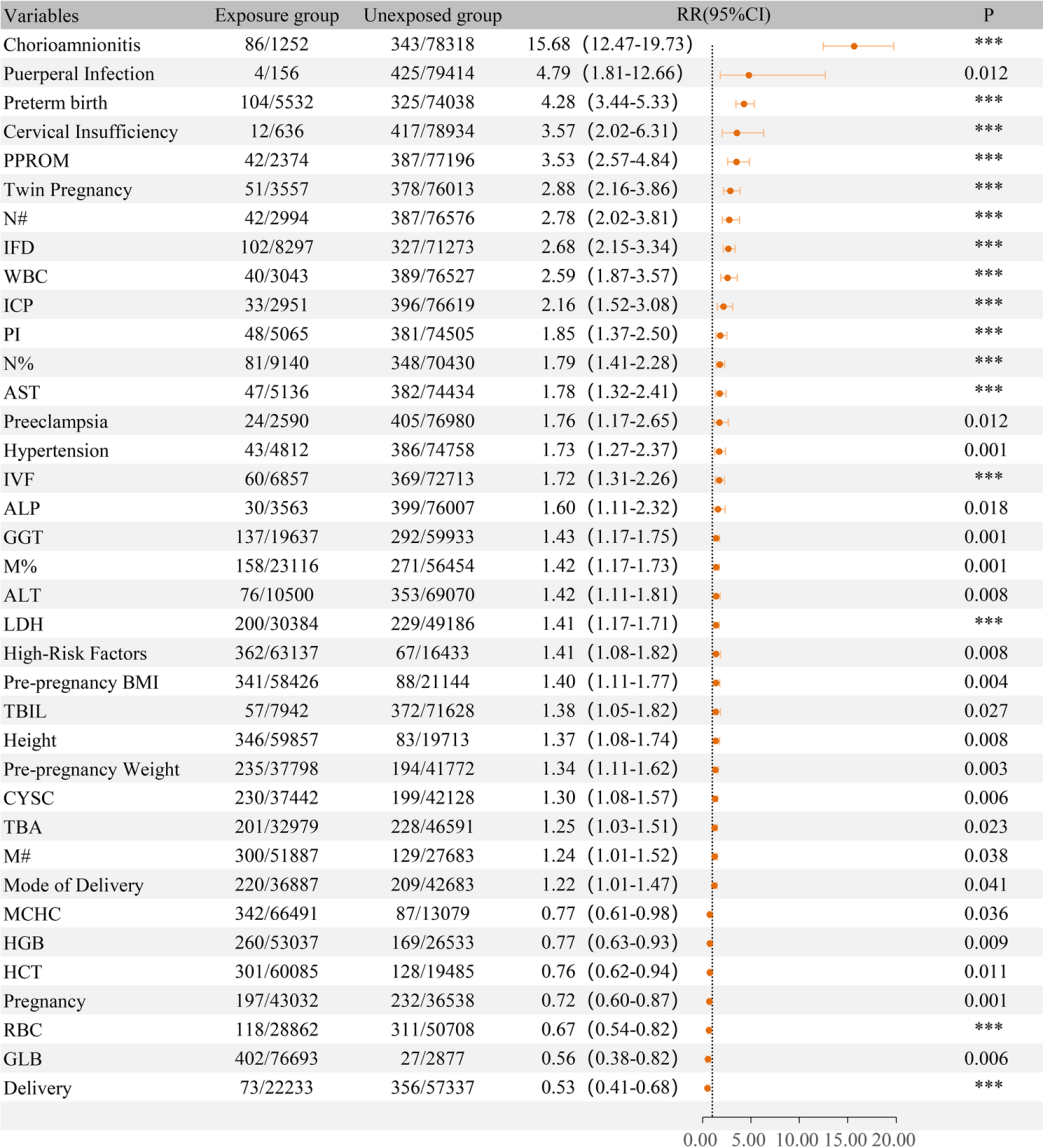
Univariate analysis of maternal factors associated with the risk of early-onset sepsis (EOS). PPROM, preterm premature rupture of membranes. IFD, Intrauterine fetal distress. PI, placenta invasion. ICP, intrahepatic cholestasis of pregnancy. IVF, in vitro fertilization. WBC, white blood cell count. RBC, red blood cell count. HGB, hemoglobin. HCT, hematocrit. MCHC, mean corpuscular hemoglobin concentration. GLB, globulin. TBIL, total bilirubin. TBA, total bile acids. ALT, alanine aminotransferase. AST, aspartate aminotransferase. ALP, alkaline phosphatase. GGT, gamma-glutamyl transferase. LDH, lactate dehydrogenase. CYSC, cystatin C. N%, neutrophil percentage. M%, monocyte percentage. N#, neutrophil absolute count. M#, monocyte absolute count

Univariate analysis identified several maternal categorical variables significantly associated with EOS risk. The following conditions were found to increase EOS risk significantly: premature rupture of membranes (PPROM) (RR = 3.53, 95% CI 2.57–4.84, *P* < 0.001), preterm birth (RR = 4.28, 95% CI 3.44–5.33, *P* < 0.001), intrauterine fetal distress (IFD) (RR = 2.68, 95% CI 2.15–3.34, *P* < 0.001), placental implantation (PI) abnormalities (RR = 1.85, 95% CI 1.37–2.50, *P* < 0.001), intrahepatic cholestasis of pregnancy (ICP) (RR = 2.16, 95% CI 1.52–3.08, *P* < 0.001), hypertension (RR = 1.73, 95% CI 1.27–2.37, *P* = 0.001), preeclampsia (RR = 1.76, 95% CI 1.17–2.65, *P* = 0.012), cervical insufficiency (RR = 3.57, 95% CI 2.02–6.31, *P* < 0.001), maternal chorioamnionitis (RR = 15.68, 95% CI 12.47–19.73, *P* < 0.001), puerperal infection (RR = 4.79, 95% CI 1.81–12.66, *P* = 0.012), twin pregnancy (RR = 2.88, 95% CI 2.16–3.86, *P* < 0.001), mode of delivery (RR = 1.22, 95% CI 1.01–1.47, *P* = 0.041), high-risk pregnancy factors (RR = 1.41, 95% CI 1.08–1.82, *P* = 0.008), and in vitro fertilization (IVF) (RR = 1.72, 95% CI 1.31–2.26, *P* < 0.001). In contrast, a history of pregnancy (RR = 0.72, 95% CI 0.60–0.87, *P* = 0.001) and a history of delivery (RR = 0.53, 95% CI 0.41–0.68, *P* < 0.001) were associated with a significantly lower risk of EOS. Other factors, such as nuchal cord and hepatitis B infection, showed no significant association with the risk of EOS (*P* > 0.05) (Fig. 2 and supplementary Table 1).

### The maternal continuous variables associated with the risk of EOS

To assess the impact of maternal continuous variables on EOS, we first determined the optimal cut-off thresholds derived from receiver operating characteristic curve analysis for all continuous indicators (see Supplementary Table 2) and dichotomized them accordingly. The results demonstrated that elevated maternal levels of WBC (RR = 2.59, 95% CI 1.87–3.57, *P* < 0.001), AST (RR = 1.78, 95% CI 1.32–2.41, *P* < 0.001), alkaline phosphatase (ALP) (RR = 1.60, 95% CI 1.11–2.32, *P* = 0.018), as well as higher values of pre-pregnancy weight, height, pre-pregnancy BMI, neutrophil percentage (N%), monocyte percentage (M%), N#, M#, total bilirubin (TBIL), total bile acids (TBA), ALT, gamma-glutamyl transferase (GGT), lactate dehydrogenase (LDH), and cystatin C (CYSC) were significantly associated with increased risk of EOS. Conversely, increased levels of globulin (GLB) (RR = 0.56, 95% CI 0.38–0.82, *P* = 0.006), hemoglobin (HGB) (RR = 0.77, 95% CI 0.63–0.93, *P* = 0.009), red blood cell count (RBC), hematocrit (HCT), and mean corpuscular hemoglobin concentration (MCHC) were significantly associated with reduced risk of EOS (Fig. 2).

**Fig. 3:**
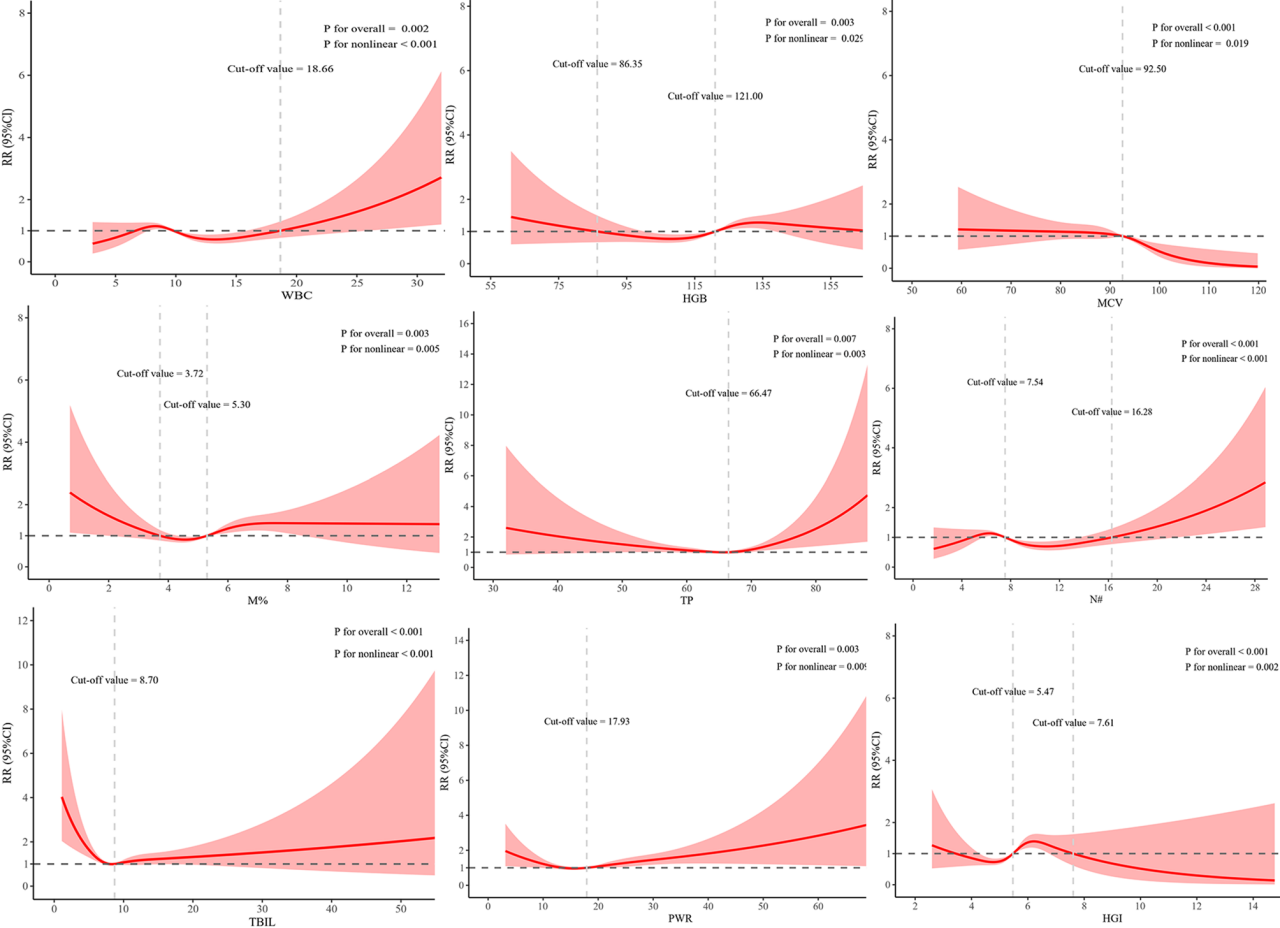
Restricted cubic spline (RCS) analyses of maternal continuous variables associated with the risk of early-onset sepsis (EOS). WBC, White blood cell count. HGB, hemoglobin. MCV, mean corpuscular volume. MCH, mean corpuscular hemoglobin. M%, monocyte percentage. N#, neutrophil absolute count. TP, total protein. TBIL, total bilirubin. PWR, platelet to white cell ratio. SIRI, systemic inflammation response Index. HGI, hemoglobin to glycerinated hemoglobin index

Restricted cubic spline analysis was further employed to evaluate potential non-linear associations and enhance robustness. Significant non-linear relationships were observed between EOS risk and the following maternal variables: WBC (*P* < 0.001), HGB (*P* = 0.029), mean corpuscular volume (MCV) (*P* = 0.019), mean corpuscular hemoglobin (MCH) (*P* = 0.039), M% (*P* = 0.005), N# (*P* < 0.001), total protein (TP) (*P* = 0.003), TBIL (*P* < 0.001), UREA (*P* = 0.034), PWR (*P* = 0.009), SIRI (*P* = 0.045), and HGI (*P* = 0.002). All of these variables also showed statistically significant overall associations with EOS (WBC: *P* = 0.002; HGB: *P* = 0.003; MCV: *P* < 0.001; MCH: *P* = 0.015; M%: *P* = 0.003; N#: *P* < 0.001; TP: *P* = 0.007; TBIL: *P* < 0.001; PWR: *P* = 0.003; SIRI: *P* = 0.008; HGI: *P* < 0.001). Specifically, the risk of EOS increased substantially when maternal values exceeded or fell below the following thresholds: WBC > 18.66 × 10⁹/L, HGB < 86.35 g/L, MCV < 92.50 fL, MCH < 31.50 pg, M% < 3.72% or > 5.30%, N# >16.28 × 10⁹/L, UREA > 11.89 mmol/L, PWR > 17.93, and SIRI > 14.16. Additionally, TP and TBIL exhibited U-shaped associations with EOS risk, with the lowest predicted risk observed at TP = 66.47 g/L and TBIL = 8.70 µmol/L (Fig. 3 and supplementary Table 3).

### Selection of significant variables and model construction

**Fig. 4:**
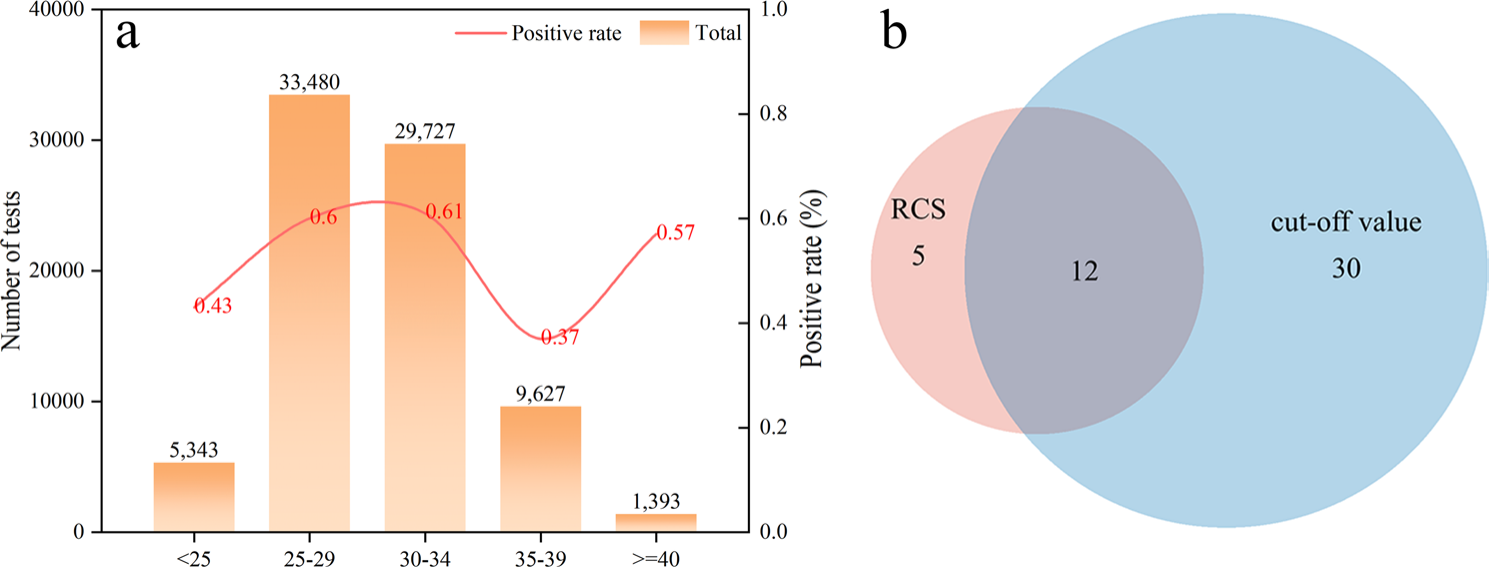
Relationship between maternal factors and the risk of early-onset sepsis (EOS). **a** EOS incidence by maternal age groups. The bar chart illustrates the proportion of EOS cases across different maternal age categories (< 25, 25–29, 30–34, 35–39, and ≥ 40 years). The highest EOS risk was observed among neonates born to mothers aged 25–34 years. **b** Venn diagram showing the intersection of significantly associated maternal continuous variables identified by two approaches: cut-off based univariate analysis and restricted cubic spline (RCS) modeling. Variables identified by both methods were considered robust predictors and selected for subsequent multivariable modeling

To identify robust predictors of EOS, a Venn diagram was employed to visualize the overlap between variables identified by two independent approaches: (1) univariate analysis based on dichotomized continuous variables using optimal cut-off values, and (2) RCS modeling. A total of 12 overlapping variables were identified, including WBC, RBC, HGB, HCT, M%, N#, GLB, TBIL, SIRI, DNLR, HGI, and PWR (Fig. 4b and supplementary Table 4). These variables demonstrated consistent associations with EOS across both methods and were selected for further modeling. Additionally, maternal categorical variables that showed significant associations in prior univariate analyses were also included as candidates for model development.

Given potential multicollinearity among the selected variables, collinearity diagnostics were performed using variance inflation factors (VIFs) and tolerance statistics. Variables exhibiting high collinearity were excluded to enhance model stability and interpretability (Supplementary Table 5). The remaining predictors were incorporated into a multivariable Poisson regression model (Model 1), adjusted for maternal age and year of neonatal admission (Table 2). From Model 1, maternal factors significantly associated with EOS risk were retained in the final predictive model (Model 2). Among these, chorioamnionitis (IRR = 13.16, 95% CI: 10.20–16.98, *P* < 0.001) and puerperal infection (IRR = 3.91, 95% CI: 1.56–9.83, *P* = 0.004) were associated with the highest risk. Neonates born to mothers with twin pregnancies had significantly higher EOS risk compared to singletons (IRR = 1.65, 95% CI 1.15–2.35, *P* = 0.006). Delivery mode was also associated with the risk of EOS: cesarean section significantly reduced the likelihood of EOS (IRR = 0.67, 95% CI 0.54–0.82, *P* < 0.001). Additionally, maternal GLB levels were inversely associated with EOS risk (IRR = 0.58, 95% CI 0.40–0.85, *P* = 0.005), whereas elevated PWR (IRR = 1.33, 95% CI 1.05–1.68, *P* = 0.018), HGI (IRR = 1.72, 95% CI 1.42–2.08, *P* < 0.001), and M% (IRR = 1.43, 95% CI 1.17–1.75, *P* < 0.001) were associated with increased risk. Other maternal conditions such as preterm birth, IFD, PI, ICP, and cervical insufficiency were also found to be independently associated with elevated EOS risk (Table 3).

**Table 2:** Model 1: a multivariable Poisson regression model was used to evaluate the association between maternal factors and the risk of EOS

Variables	IRR	z	95% CI		*P*
			ll	ul	
Age	0.99	− 0.70	0.97	1.02	0.487
Year	0.92	− 2.91	0.87	0.97	0.004
PPROM	0.99	− 0.07	0.67	1.46	0.944
Preterm birth	3.40	7.72	2.49	4.65	0.000
IFD	2.18	6.12	1.70	2.80	0.000
PI	1.47	2.43	1.08	1.99	0.015
ICP	1.67	2.77	1.16	2.40	0.006
Hypertension	1.33	1.20	0.83	2.11	0.232
Preeclampsia	0.89	− 0.39	0.49	1.62	0.699
Cervical Insufficiency	1.91	2.12	1.05	3.48	0.034
Chorioamnionitis	11.38	17.77	8.70	14.88	0.000
Puerperal infection	3.56	2.72	1.43	8.89	0.006
Twin pregnancy	1.60	2.19	1.05	2.44	0.029
Mode of delivery	0.66	− 4.05	0.53	0.80	0.000
WBC	1.25	1.05	0.82	1.90	0.296
HGB	0.95	− 0.44	0.75	1.20	0.660
M%	1.52	3.92	1.23	1.87	0.000
GLB	0.59	− 2.73	0.40	0.86	0.006
SIRI	1.33	1.58	0.93	1.89	0.114
DNLR	1.22	1.30	0.90	1.64	0.195
HGI	1.65	4.26	1.31	2.08	0.000
PWR	1.43	2.88	1.12	1.82	0.004
TBIL	1.31	1.90	0.99	1.73	0.057
High-risk factors	1.20	1.33	0.92	1.57	0.185
IVF	0.91	− 0.49	0.63	1.32	0.624
Pregnancy	0.88	− 1.14	0.70	1.10	0.256
Delivery	0.78	− 1.62	0.57	1.05	0.105

IRR, Incidence rate ratio; 95%CI, 95% confidence interval; ll, lower limit; ul, upper limit; PPROM, preterm premature rupture of membranes; IFD, intrauterine fetal distress; PI, placenta invasion; ICP, intrahepatic cholestasis of pregnancy; IVF, in vitro fertilization; WBC, white blood cell count; HGB, hemoglobin; M%, monocyte percentage; GLB, globulin; TBIL, total bilirubin; N%, neutrophil percentage; N#, neutrophil absolute count; M#, monocyte absolute count; PWR, platelet to white cell ratio; SIRI, systemic inflammation response index; DNLR, derived neutrophil to lymphocyte ratio; HGI, hemoglobin to glycerinated hemoglobin index

**Table 3:** A multivariable Poisson regression (Model 2) and logistic regression (sensitivity analysis) adjusted for key maternal covariates to examine their association with the incidence of EOS

Variables	Model 2					Sensitivity analysis				
	IRR	z	95% CI		*P*	IOR	z	95% CI		*P*
			ll	ul				ll	ul	
Age	0.98	− 1.64	0.96	1.00	0.102	0.98	− 1.47	0.96	1.01	0.140
Year	0.92	− 2.94	0.87	0.97	0.003	0.92	− 3.00	0.87	0.97	0.003
Preterm birth	3.37	9.13	2.60	4.38	< 0.001	3.51	9.40	2.70	4.55	< 0.001
IFD	2.30	6.62	1.80	2.94	< 0.001	2.35	6.72	1.83	3.02	< 0.001
PI	1.46	2.44	1.08	1.98	0.015	1.49	2.45	1.08	2.06	0.014
ICP	1.78	3.16	1.24	2.55	0.002	1.82	3.11	1.25	2.65	0.002
Cervical Insufficiency	1.85	2.04	1.02	3.33	0.041	1.96	2.12	1.05	3.66	0.034
Chorioamnionitis	13.16	19.82	10.20	16.98	< 0.001	14.74	19.94	11.31	19.20	< 0.001
Puerperal Infection	3.91	2.90	1.56	9.83	0.004	4.11	2.70	1.47	11.47	0.007
Twin pregnancy	1.65	2.75	1.15	2.35	0.006	1.68	2.91	1.18	2.38	0.004
Mode of delivery	0.67	− 3.90	0.54	0.82	< 0.001	0.66	− 3.75	0.53	0.82	< 0.001
GLB	0.58	− 2.79	0.40	0.85	0.005	0.57	− 2.73	0.38	0.85	0.006
PWR	1.33	2.36	1.05	1.68	0.018	1.34	2.34	1.05	1.70	0.019
HGI	1.72	5.62	1.42	2.08	< 0.001	1.74	5.56	1.43	2.12	< 0.001
M%	1.43	3.54	1.17	1.75	< 0.001	1.45	3.57	1.18	1.77	< 0.001

EOS, early-onset sepsis; IRR, incidence rate ratio; 95%CI, 95% confidence interval; ll, lower limit; ul, upper limit; IFD, intrauterine fetal distress; PI, placenta invasion; ICP, intrahepatic cholestasis of pregnancy; M%, monocyte percentage; GLB, globulin; PWR, platelet to white cell ratio; HGI, hemoglobin to glycerinated hemoglobin index

### Model performance evaluation and visualization

**Fig. 5:**
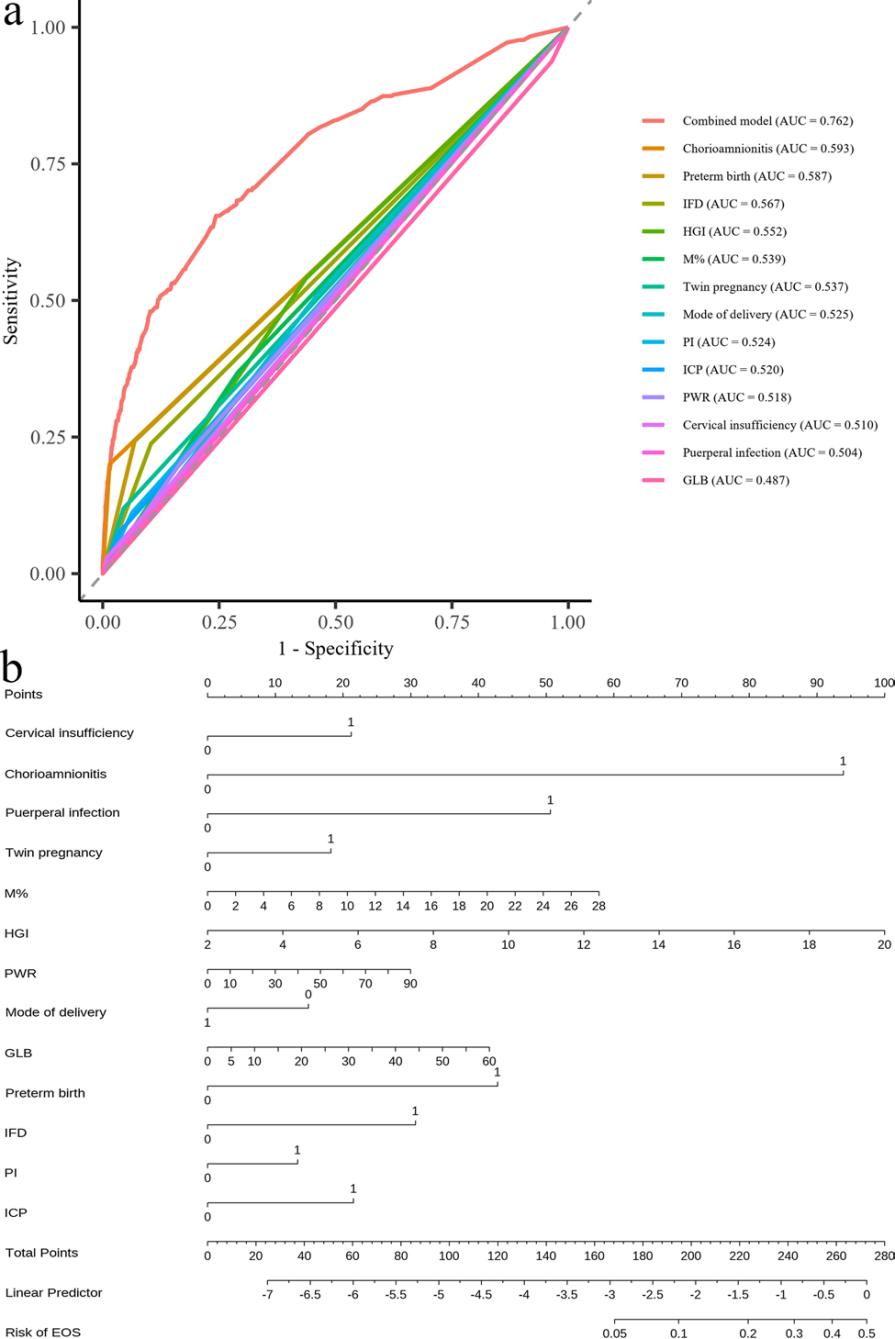
Development of a maternal factor-based prediction model for early-onset sepsis (EOS). **a** ROC. **b** nomogram. IFD, intrauterine fetal distress. HGI, hemoglobin to glycerinated hemoglobin index. M%, monocyte percentage. PI, placenta invasion. ICP, intrahepatic cholestasis of pregnancy. PWR, platelet to white cell ratio. GLB, globulin

To evaluate the predictive performance of the model 2, a receiver operating characteristic curve was plotted and the AUC was calculated. The model demonstrated good discriminative ability for predicting EOS, with an AUC of 0.762 (*P* < 0.001), indicating good discriminative ability for EOS (Fig. 5a). Based on the multivariable Poisson regression model 2, a nomogram was constructed to facilitate individualized risk prediction and improve clinical applicability. The nomogram incorporated 13 maternal predictors. Each variable was assigned a weighted score proportional to its contribution to the risk of EOS. By summing the individual scores, clinicians can estimate a newborn’s predicted probability of developing EOS using the scale at the bottom of the nomogram. This tool offers a practical and visual approach for early risk stratification and decision-making in neonatal care (Fig. 5b).

### Sensitivity analysis

**Fig. 6:**
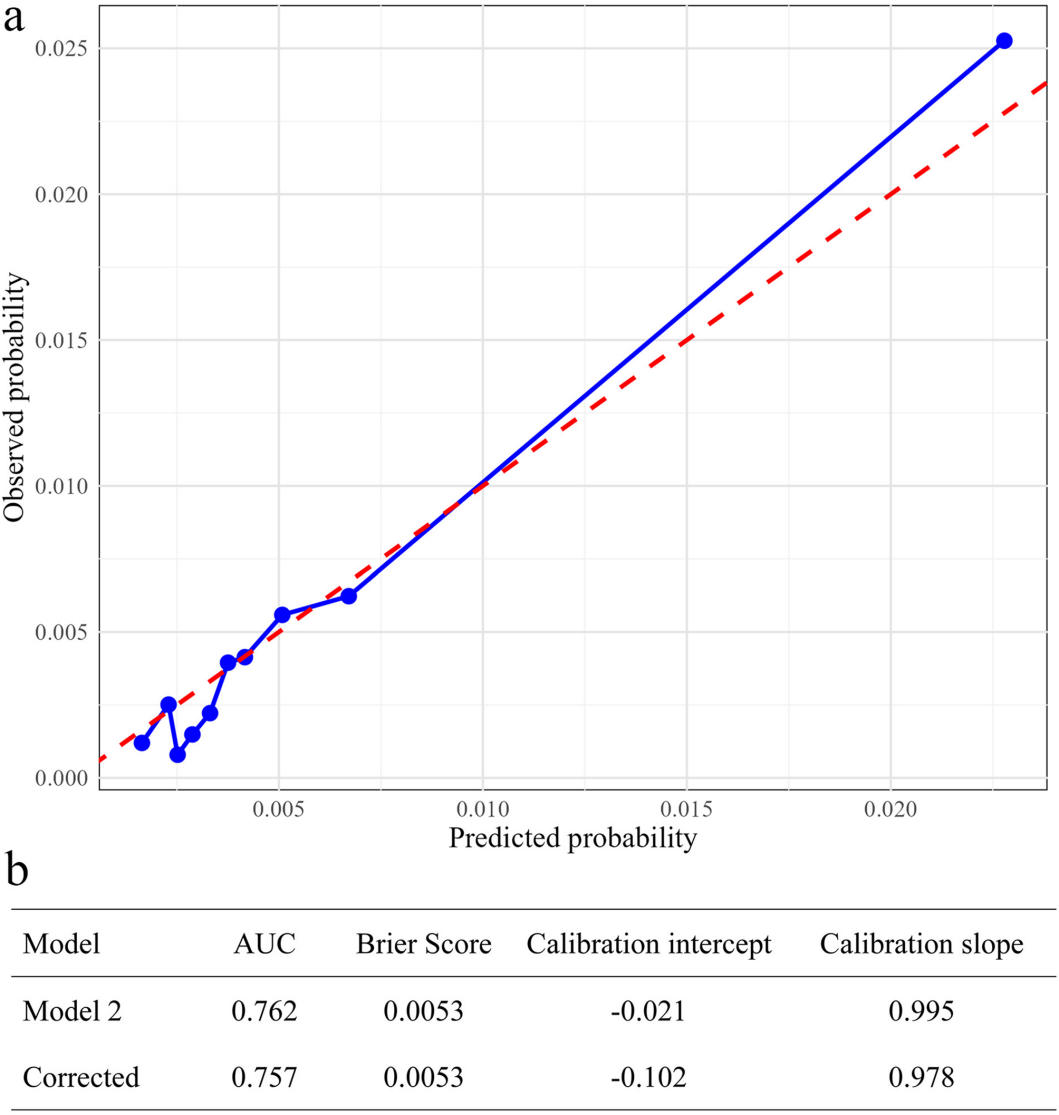
Performance evaluation of the model. **a**, the bootstrap-corrected calibration plot. **b**, bootstrapping result

To ensure the robustness of our findings, we conducted a multivariate logistic regression analysis using the variables included in Model 2. The results were consistent with those obtained from Poisson regression. (all *P* values < 0.05) (Table 3). In the bootstrap results, the apparent performance metrics indicated that the model had good discriminative ability for EOS (AUC = 0.762), a Brier score of 0.0053, a calibration intercept close to zero (− 0.021), and a calibration slope near one (0.995). After bootstrap correction, the model’s performance remained stable (AUC = 0.757, Brier score = 0.0053, calibration intercept = − 0.102, calibration slope = 0.978), indicating consistent performance across resampled datasets, low risk of overfitting, and robust and reliable predictive capability (Fig. 6b). Furthermore, the bootstrap-corrected calibration plot demonstrated a high concordance between predicted probabilities and observed risk, providing additional support for the model’s clinical applicability for individualized EOS risk assessment (Fig. 6a).

## Discussion

Neonatal sepsis is generally classified into EOS and LOS [12]. EOS is typically caused by vertical transmission of pathogens from the mother, occurring either before or during delivery [13]. The disease progresses rapidly and is often characterized by nonspecific and atypical clinical manifestations, which may lead to serious complications or even neonatal death [14, 15]. Therefore, there is an urgent clinical need to establish effective preventive strategies to reduce the incidence of neonatal sepsis. In China, studies focusing on the development of predictive models for EOS remain limited, especially those incorporating maternal factors [4]. Although certain inflammatory markers and clinical signs are currently used in clinical practice to assess the risk of neonatal sepsis, a comprehensive, visualized, and quantitative prediction model is still lacking. which hinders early diagnosis and timely intervention [16]. The present study aimed to investigate the potential association between maternal factors and the risk of EOS, with the intention of developing a predictive model that may assist in estimating the likelihood of EOS before or shortly after delivery. Such a model could potentially support earlier identification and intervention, thereby contributing to improved neonatal outcomes. In this study, we conducted a comprehensive analysis of the association between maternal factors and the risk of EOS in neonates, using a large-scale retrospective cohort of 79,570 successfully matched mother–infant pairs. A predictive model was developed using multivariable Poisson regression, which demonstrated good discriminatory performance for EOS risk (AUC = 0.762). The model was further visualized as a nomogram incorporating 13 maternal-related predictors, enabling individualized risk assessment for EOS.

This study found that neonates born to mothers aged 25–34 years had the highest risk of EOS, with an incidence of 0.60–0.61%. This elevated risk may be attributable to higher birth density and an increased incidence of pregnancy complications, such as chorioamnionitis, in this maternal age group [17]. Several perinatal maternal factors were significantly associated with the occurrence of EOS. Chorioamnionitis (IRR = 16.16, 95% CI 10.20–16.98) and puerperal infection (IRR = 3.91, 95% CI 1.56–9.83) were identified as the strongest predictors, likely due to their direct contribution to intrauterine infection [18]. From a mechanistic perspective, maternal genital tract pathogens (commonly Group B Streptococcus, *Escherichia coli*, and Ureaplasma/Mycoplasma spp.) can ascend through the cervix to the chorion–amnion, leading to chorioamnionitis and contamination of the amniotic fluid [19–21]. The fetus may aspirate or swallow the contaminated fluid, or acquire pathogens via skin and mucosal contact, establishing colonization or invasion before or immediately after birth [22]. Inflammation also increases the permeability of the placental barrier, and in the presence of maternal bacteremia, hematogenous transplacental transmission to the fetus may occur [21]. At the maternal–fetal interface, chorioamnionitis activates innate immune pathways in the placenta and membranes via TLR2/TLR4 recognition of Gram-positive or Gram-negative pathogen-associated molecular patterns (PAMPs), triggering the MyD88–NF-κB signaling cascade. This leads to the release of pro-inflammatory cytokines such as IL-1β, IL-6, and TNF-α, neutrophil infiltration, and complement activation [23]. These processes may contribute to fetal inflammatory response syndrome and increase neonatal susceptibility to EOS [24]. Additionally, cervical insufficiency, intrauterine fetal distress, preterm birth, premature rupture of membranes, and intrahepatic cholestasis of pregnancy were all significantly associated with increased risk of EOS, highlighting the critical role of adverse intrauterine environments and intrapartum complications [25]. Twin pregnancies were also associated with a higher risk of EOS, possibly due to higher rates of preterm delivery and more frequent clinical interventions [26]. Moreover, vaginal delivery in the setting of maternal infection increases intrapartum exposure to pathogens, whereas elective cesarean delivery may reduce risk of EOS (IRR = 0.67, 95% CI 0.54–0.82) [27].

In terms of maternal laboratory parameters, this study identified several hematological and biochemical markers significantly associated with the risk of EOS. Elevated levels of HGI, M%, PWR, and SIRI, along with reduced levels of GLB and HGB, were associated with increased risk of EOS. These findings suggest that maternal inflammatory status, immune alterations, and hepatic function may play critical roles in determining fetal susceptibility to infection [3, 14]. Further analysis using restricted cubic splines revealed nonlinear associations between several maternal biomarkers (e.g., WBC, HGB, GLB, TP, and TBIL) and the risk of EOS, highlighting potential threshold effects and offering quantitative insights for clinical monitoring. Specifically, maternal WBC counts >18.66 × 10⁹/L and/or PWR values >17.93 were significantly associated with an increased risk of EOS. The WBC threshold identified in this study exceeds the upper limit of the reference range for pregnant women in most guidelines (15 × 10⁹/L), suggesting that more pronounced leukocytosis may be particularly relevant to EOS risk. Leukocytosis during pregnancy may indicate intrauterine infection, such as chorioamnionitis, or ongoing maternal inflammation [28]. Through the action of inflammatory mediators and proteolytic enzymes, these conditions can damage fetal membranes and epithelial integrity, thereby weakening the physical and chemical barrier functions of the fetal or neonatal mucosa and increasing the risk of bacterial translocation into the bloodstream [29, 30]. Similarly, the PWR threshold (17.93) has not been explicitly included in current obstetric or neonatal guidelines, highlighting the need for further validation before widespread adoption. Nevertheless, PWR, an inflammation-sensitive ratio, may reflect dysregulation in the immune–coagulation axis, potentially driven by relative leukopenia or thrombocytosis, both of which could disrupt maternal–fetal immune balance and increase EOS risk [29]. These thresholds may serve as early warning indicators in high-risk pregnancies, prompting closer perinatal surveillance. Maternal TP and TBIL levels also exhibited a U-shaped relationship with EOS risk. Low TP or TBIL levels may indicate maternal malnutrition, impaired hepatic function, or immunoglobulin deficiency, resulting in suboptimal intrauterine immune protection [31]. Conversely, elevated TP or TBIL levels may be associated with cholestasis, hepatocellular injury, or systemic inflammation, which may compromise placental function or alter microbial colonization in utero, thereby facilitating EOS development [4, 31].

In this study, we explored the potential association between maternal factors and the risk of EOS in neonates. Maternal factors may provide a valuable approach for estimating EOS risk, as they are typically available before or at delivery, allowing early identification of neonates who could benefit from closer monitoring or timely interventions. Compared with neonatal clinical signs and laboratory parameters, maternal factors are generally more objective, easily obtainable, noninvasive, and pose no risk to the newborn, suggesting their potential applicability across diverse clinical settings. Moreover, maternal indicators may offer indirect information about the intrauterine environment, which could help elucidate mechanisms contributing to EOS development.

To ensure the robustness of our findings, we conducted a sensitivity analysis using multivariable logistic regression with the variables from Model 2. The results were consistent with those obtained from the Poisson regression model, further supporting the reliability of our conclusions. Integrating multiple statistical approaches, including cut-off value–based grouping, restricted cubic spline modeling, Venn diagram–based variable selection, and multivariable regression analysis, enhanced the scientific rigor of variable selection and improved model stability. The prevalence of certain maternal conditions, such as ICP, and healthcare practices, such as cesarean section rates, may vary across regions. For instance, in Western countries (e.g., North America and Europe), the incidence of ICP is generally low, whereas in some Asian regions (including certain provinces in China), parts of South America, or specific populations in Northern Europe, it can reach 1–15% or even higher [32]. The prevalence observed in one region may not be directly generalizable to other settings. Consequently, if our model were to be applied in different regions, particularly outside China, external validation in diverse populations would likely be advisable.

This study may be subject to a certain degree of misclassification bias. First, some maternal conditions, laboratory parameters, and neonatal EOS events could have been incorrectly classified due to incomplete or erroneous electronic medical record entries, potentially influencing the results. Second, since the data were collected from specific regions and healthcare institutions, the study population may not fully represent other settings or populations, which could limit the generalizability of the findings. Finally, maternal antibiotic exposure prior to delivery was not successfully captured during data collection. The use of antenatal antibiotics carries both potential benefits and risks, and usage patterns can vary across regions, hospitals, and time periods. As such, antibiotic exposure represents a potential predictive factor, and its omission may reduce the discriminative ability of the model. Therefore, if this model is to be applied in other regions, particularly outside China, further external validation would be advisable. EOS risk predictions based on this model should be interpreted with consideration of local clinical practices and context.

In conclusion, this study developed a maternal factor–based prediction model for neonatal EOS, demonstrating favorable discriminative performance and potential clinical utility. The nomogram, as a visualized risk assessment tool, enables individualized prediction of EOS risk and offers a novel approach for the early identification and precise management of high-risk neonates during the perinatal period. Moving forward, to ensure the model’s predictive performance and wider applicability, prospective validation in diverse clinical settings should be prioritized, along with exploration of its integration into routine perinatal care workflows to support timely EOS risk assessment.

## Supplementary Information

Below is the link to the electronic supplementary material.

Supplementary Material 1

## Data Availability

All raw data was deposited on Zenodo [33].

## Abbreviations

EOS: Early-onset sepsis
RCS: Restricted cubic spline
LOS: Late-onset sepsis
WBC: White blood cell
PLT: Platelet
RBC: Red blood cell count
HGB: Hemoglobin
HCT: Hematocrit
MCV: Mean corpuscular volume
MCH: Mean corpuscular hemoglobin
MCHC: Mean corpuscular hemoglobin concentration
RDW: Red cell distribution width
RDWSD: Red cell distribution width (standard deviation)
L%: Lymphocyte percentage
E%: Eosinophil percentage
L#: Lymphocyte absolute count
E#: Eosinophil absolute count
N%: Neutrophil percentage
N#: Neutrophil absolute count
M#: Monocyte absolute count
M%: Monocyte percentage
B%: Basophil percentage
B#: Basophil absolute count
TP: Total protein
GLB: Globulin
ALB: Albumin
A/G: Albumin/globulin ratio
PA: Prealbumin
TBIL: Total bilirubin
DBIL: Direct bilirubin
IBIL: Indirect bilirubin
ALT: Alanine aminotransferase
AST: Aspartate aminotransferase
ALP: Alkaline phosphatase
GGT: Gamma-glutamyl transferase
LDH: Lactate dehydrogenase
CR: Creatinine
UA: Uric acid
CYSC: Cystatin C
HIS: Hospital information system
LIS: Laboratory information system
IQR: Interquartile range
VIF: Inflation factor
CIs: Confidence intervals
IRR: Incidence rate ratio
PROM: Premature rupture of membranes
TPL: Threatened preterm labor
FGR: Fetal growth restriction
GBS: Group B streptococcus
PHM: Pregnancy with high myopia
PUF: Pregnancy with uterine fibroids
Pregnancy-T: Pregnancy with thrombocytopenia
Postpartum-A: Postpartum anemia
Postpartum-H: Postpartum hemorrhage
AOH: Abnormal obstetric history
ACH: Alcohol consumption history
ll: Lower limit
ul: Upper limit
IFD: Intrauterine fetal distress
PI: Placenta invasion
ICP: Intrahepatic cholestasis of pregnancy
PWR: Platelet to white cell ratio
HGI: Hemoglobin to glycerinated hemoglobin index
NLR: Neutrophil to lymphocyte ratio
MLR: Monocyte to lymphocyte ratio
NMLR: (Neutrophil + monocyte) to lymphocyte ratio
SIRI: Systemic inflammation response index
SII: Systemic immune-inflammation index
DNLR: Derived neutrophil to lymphocyte ratio
SLR: AST to ALT ratio
UCR: UREA to creatinine ratio
PNI: Prognostic nutrition index

## Code availability

No custom codes were used in this study.
