# Supplementary material for: Maternal predictors of early-onset sepsis in neonates: a multicenter retrospective cohort study and risk prediction model
Source: J Transl Med. 2025 Oct 16;23:1114. doi: 10.1186/s12967-025-07154-2 (PMC12532861; doi:10.1186/s12967-025-07154-2)
Supplement: Supplementary file 1 — Supplementary Material 1 [file 12967_2025_7154_MOESM1_ESM.docx]

Supplementary table 1. Maternal factors that were not significantly associated with EOS in univariable analysis.

| Variables | Exposure group | | | Unexposed group | | | RR(95%CI) | P |
| --- | --- | --- | --- | --- | --- | --- | --- | --- |
|  | + | - | Total | + | - | Total |  |  |
| PROM | 98 | 18791 | 18889 | 331 | 60350 | 60681 | 0.95（0.76-1.19） | 0.669 |
| TPL | 2 | 686 | 688 | 427 | 78455 | 78882 | 0.54（0.13-2.15） | 0.396 |
| FGR | 7 | 1387 | 1394 | 422 | 77754 | 78176 | 0.93（0.44-1.96） | 0.895 |
| Nuchal Cord | 109 | 22008 | 22117 | 320 | 57133 | 57453 | 0.89（0.71-1.10） | 0.268 |
| Oligohydramnios | 55 | 9143 | 9198 | 374 | 69998 | 70372 | 1.13（0.85-1.49） | 0.410 |
| Placenta Previa | 13 | 1544 | 1557 | 416 | 77597 | 78013 | 1.57（0.90-2.71） | 0.127 |
| Hypothyroidism | 44 | 7475 | 7519 | 385 | 71666 | 72051 | 1.10（0.80-1.50） | 0.558 |
| PHM | 20 | 4585 | 4605 | 409 | 74556 | 74965 | 0.80（0.51-1.25） | 0.319 |
| PUF | 16 | 3000 | 3016 | 413 | 76141 | 76554 | 0.98（0.60-1.62） | 0.978 |
| Pregnancy-T | 10 | 2339 | 2349 | 419 | 76802 | 77221 | 0.79（0.42-1.47） | 0.462 |
| Hepatitis B | 12 | 1594 | 1606 | 417 | 77547 | 77964 | 1.40（0.79-2.48） | 0.261 |
| Thalassemia | 21 | 3050 | 3071 | 408 | 76091 | 76499 | 1.28（0.83-1.99） | 0.270 |
| Postpartum-A | 62 | 9818 | 9880 | 367 | 69323 | 69690 | 1.19（0.91-1.56） | 0.205 |
| Postpartum-H | 13 | 2549 | 2562 | 416 | 76592 | 77008 | 0.94（0.54-1.63） | 0.856 |
| GBS | 38 | 5339 | 5377 | 391 | 73802 | 74193 | 1.34（0.96-1.87） | 0.092 |
| AOH | 169 | 34084 | 34253 | 260 | 45057 | 45317 | 0.86（0.71-1.04） | 0.125 |
| Smoking History | 10 | 2466 | 2476 | 419 | 76675 | 77094 | 0.74（0.40-1.39） | 0.357 |
| ACH | 55 | 10661 | 10716 | 374 | 68480 | 68854 | 0.95（0.71-1.25） | 0.705 |
| MCV | 1 | 558 | 559 | 428 | 78583 | 79011 | 0.33（0.05-2.35） | 0.243 |
| MCH | 1 | 446 | 447 | 428 | 78695 | 79123 | 0.41（0.06-2.94） | 0.393 |
| RDWSD | 428 | 78624 | 79052 | 1 | 517 | 518 | 2.81（0.40-19.92） | 0.291 |
| RDW | 287 | 49367 | 49654 | 142 | 29774 | 29916 | 1.22（1.00-1.49） | 0.053 |
| L% | 15 | 2057 | 2072 | 414 | 77084 | 77498 | 1.36（0.81-2.26） | 0.254 |
| E% | 56 | 8994 | 9050 | 373 | 70147 | 70520 | 1.17（0.88-1.55） | 0.275 |
| L# | 428 | 78932 | 79360 | 1 | 209 | 210 | 1.13（0.16-8.02） | 0.992 |
| E# | 165 | 29978 | 30143 | 264 | 49163 | 49427 | 1.03（0.84-1.24） | 0.802 |
| PA | 428 | 79130 | 79558 | 1 | 11 | 12 | 0.07（0.01-0.42） | 0.065 |
| TP | 388 | 73449 | 73837 | 41 | 5692 | 5733 | 0.74（0.53-1.01） | 0.068 |
| ALB | 422 | 77910 | 78332 | 7 | 1231 | 1238 | 0.95（0.45-2.01） | 0.854 |
| A/G | 427 | 78981 | 79408 | 2 | 160 | 162 | 0.44（0.11-1.73） | 0.276 |
| DBIL | 158 | 26760 | 26918 | 271 | 52381 | 52652 | 1.14（0.94-1.39） | 0.190 |
| IBIL | 51 | 7838 | 7889 | 378 | 71303 | 71681 | 1.23（0.92-1.64） | 0.177 |
| UREA | 28 | 4427 | 4455 | 401 | 74714 | 75115 | 1.18（0.80-1.73） | 0.399 |
| CR | 250 | 43496 | 43746 | 179 | 35645 | 35824 | 1.14（0.94-1.39） | 0.169 |
| UA | 92 | 14324 | 14416 | 337 | 64817 | 65154 | 1.23（0.98-1.55） | 0.078 |

EOS, Early-onset sepsis. PROM, Premature rupture of membranes. TPL, Threatened preterm labor. FGR, Fetal growth restriction. GBS, Group B streptococcus. MCV, Mean corpuscular volume. MCH, Mean corpuscular hemoglobin. RDW, Red cell distribution width. RDWSD, Red cell distribution width (standard deviation). TP, Total protein. ALB, Albumin. A/G, Albumin/Globulin ratio. PA, Prealbumin. DBIL, Direct bilirubin. IBIL, Indirect bilirubin. CR, Creatinine. UA, Uric acid. PHM, Pregnancy with high myopia. PUF, Pregnancy with uterine fibroids. Pregnancy-T, Pregnancy with thrombocytopenia. Postpartum-A, Postpartum anemia. Postpartum-H, Postpartum hemorrhage. AOH, Abnormal obstetric history. ACH, Alcohol consumption history. L%, Lymphocyte percentage. E%, Eosinophil percentage. L#, Lymphocyte absolute count. E#, Eosinophil absolute count.

Supplementary table 2. Determination of the optimal cut-off value for continuous variables.

| Variables | Cut_off | Sensitivity | Specificity | Youden_Index | AUC |
| --- | --- | --- | --- | --- | --- |
| Neonatal_weight | 3515 | 0.317 | 0.737 | 0.053 | 0.463 |
| Pre_Weight | 54.95 | 0.484 | 0.583 | 0.067 | 0.538 |
| Height | 1.57 | 0.748 | 0.305 | 0.052 | 0.517 |
| Pre_BMI | 19.82 | 0.731 | 0.327 | 0.059 | 0.527 |
| WBC | 17.05 | 0.093 | 0.962 | 0.055 | 0.506 |
| PLT | 162.5 | 0.650 | 0.392 | 0.043 | 0.511 |
| RBC | 3.75 | 0.725 | 0.363 | 0.088 | 0.556 |
| HGB | 126.5 | 0.394 | 0.667 | 0.061 | 0.529 |
| HCT | 38.05 | 0.298 | 0.755 | 0.054 | 0.524 |
| MCV | 67.45 | 0.998 | 0.007 | 0.005 | 0.445 |
| MCH | 21.05 | 0.998 | 0.006 | 0.003 | 0.463 |
| MCHC | 348.5 | 0.203 | 0.836 | 0.039 | 0.518 |
| RDWSD | 35.95 | 0.998 | 0.007 | 0.004 | 0.475 |
| RDW | 13.35 | 0.669 | 0.376 | 0.045 | 0.520 |
| N% | 85.25 | 0.189 | 0.886 | 0.074 | 0.523 |
| L% | 27.75 | 0.035 | 0.974 | 0.009 | 0.468 |
| M% | 6.05 | 0.368 | 0.710 | 0.078 | 0.525 |
| E% | 1.25 | 0.131 | 0.886 | 0.017 | 0.491 |
| B% | 0.65 | 0.012 | 0.991 | 0.003 | 0.491 |
| N# | 14.7 | 0.098 | 0.963 | 0.061 | 0.507 |
| L# | 0.48 | 0.998 | 0.003 | 0.001 | 0.456 |
| M# | 0.46 | 0.681 | 0.375 | 0.056 | 0.527 |
| E# | 0.06 | 0.385 | 0.621 | 0.006 | 0.490 |
| B# | 0.01 | 0.746 | 0.291 | 0.037 | 0.501 |
| PA | 369 | 0.002 | 1.000 | 0.002 | 0.441 |
| TP | 71.5 | 0.096 | 0.928 | 0.024 | 0.47 |
| ALB | 40.5 | 0.016 | 0.984 | 0.001 | 0.446 |
| GLB | 36.5 | 0.063 | 0.964 | 0.027 | 0.498 |
| A/G | 1.85 | 0.005 | 0.998 | 0.003 | 0.457 |
| TBIL | 13.05 | 0.133 | 0.900 | 0.033 | 0.478 |
| DBIL | 1.95 | 0.368 | 0.662 | 0.03 | 0.509 |
| IBIL | 10.55 | 0.119 | 0.901 | 0.020 | 0.470 |
| TBA | 3.95 | 0.469 | 0.586 | 0.054 | 0.512 |
| ALT | 23.5 | 0.177 | 0.868 | 0.045 | 0.52 |
| AST | 32.5 | 0.110 | 0.936 | 0.045 | 0.493 |
| ALP | 265.5 | 0.070 | 0.955 | 0.025 | 0.472 |
| GGT | 18.5 | 0.319 | 0.754 | 0.073 | 0.532 |
| LDH | 180.5 | 0.466 | 0.619 | 0.085 | 0.525 |
| UREA | 5.42 | 0.065 | 0.945 | 0.010 | 0.457 |
| CR | 44.5 | 0.583 | 0.450 | 0.033 | 0.515 |
| UA | 400.5 | 0.214 | 0.819 | 0.033 | 0.503 |
| CYSC | 1.25 | 0.536 | 0.530 | 0.066 | 0.520 |
| PWR | 25.69 | 0.210 | 0.831 | 0.041 | 0.502 |
| NLR | 9.03 | 0.189 | 0.892 | 0.080 | 0.530 |
| MLR | 0.32 | 0.639 | 0.434 | 0.073 | 0.544 |
| NMLR | 9.89 | 0.175 | 0.902 | 0.077 | 0.531 |
| SIRI | 7.58 | 0.135 | 0.940 | 0.075 | 0.527 |
| SII | 1075.66 | 0.417 | 0.658 | 0.075 | 0.542 |
| DNLR | 0.95 | 0.182 | 0.864 | 0.045 | 0.490 |
| SLR | 2.59 | 0.054 | 0.965 | 0.019 | 0.470 |
| UCR | 0.1 | 0.140 | 0.871 | 0.011 | 0.450 |
| HGI | 5.59 | 0.541 | 0.563 | 0.104 | 0.543 |
| PNI | 230.01 | 1.000 | 0.001 | 0.001 | 0.442 |

WBC, White blood cell count. PLT, platelet. RBC, Red blood cell count. HGB, Hemoglobin. HCT, Hematocrit. MCV, Mean corpuscular volume. MCH, Mean corpuscular hemoglobin. MCHC, Mean corpuscular hemoglobin concentration. RDW, Red cell distribution width. RDWSD, Red cell distribution width (standard deviation). N%, Neutrophil percentage. M%, Monocyte percentage. N#, Neutrophil absolute count. M#, Monocyte absolute count. L%, Lymphocyte percentage. E%, Eosinophil percentage. L#, Lymphocyte absolute count. E#, Eosinophil absolute count. B%, Basophil percentage. B#, B%, Basophil absolute count. PA, Prealbumin. TP, Total protein. ALB, Albumin. GLB, Globulin. A/G, Albumin/Globulin ratio. TBIL, Total bilirubin. DBIL, Direct bilirubin. IBIL, Indirect bilirubin. TBA, Total bile acids. ALT, Alanine aminotransferase. AST, Aspartate aminotransferase. ALP, Alkaline phosphatase. GGT, Gamma-glutamyl transferase. LDH, Lactate dehydrogenase. CR, Creatinine. UA, Uric acid. CYSC, Cystatin C. **PWR**, Platelet to White cell Ratio. **NLR**, Neutrophil to Lymphocyte Ratio. **MLR**, Monocyte to Lymphocyte Ratio. **NMLR**, (Neutrophil + Monocyte) to Lymphocyte Ratio. **SIRI**, Systemic Inflammation Response Index. **SII**, Systemic Immune-Inflammation Index. **DNLR**, Derived Neutrophil to Lymphocyte Ratio. **SLR**, AST to ALT Ratio. **UCR,** UREA to Creatinine Ratio. **HGI**, Hemoglobin to Glycerinated Hemoglobin Index. **PNI,** Prognostic Nutrition Index

Supplementary table 3. Restricted cubic spline (RCS) analysis of continuous variables to assess their association with the risk of EOS.

| Variables | P for overall | P for nonlinear |
| --- | --- | --- |
| WBC | 0.002 | *** |
| RBC | *** | 0.028 |
| HGB | 0.003 | 0.029 |
| HCT | 0.01 | 0.101 |
| MCHC | 0.356 | 0.407 |
| N% | 0.282 | 0.156 |
| M% | 0.003 | 0.005 |
| N# | *** | *** |
| M# | 0.686 | 0.639 |
| GLB | 0.04 | 0.054 |
| TBIL | *** | *** |
| TBA | 0.586 | 0.438 |
| ALT | 0.286 | 0.636 |
| AST | 0.055 | 0.065 |
| ALP | 0.087 | 0.129 |
| GGT | 0.418 | 0.259 |
| LDH | 0.109 | 0.050 |
| CYSC | 0.241 | 0.187 |
| NLR | 0.291 | 0.348 |
| SIRI | 0.008 | 0.045 |
| DNLR | 0.019 | 0.008 |
| HGI | *** | 0.002 |
| PWR | 0.003 | 0.009 |
| MCV | *** | 0.019 |
| MCH | 0.015 | 0.039 |
| RDWSD | 0.276 | 0.77 |
| RDW | 0.113 | 0.101 |
| L% | 0.305 | 0.167 |
| E% | 0.861 | 0.816 |
| L# | 0.121 | 0.424 |
| E# | 0.942 | 0.869 |
| PA | 0.147 | 0.246 |
| TP | 0.007 | 0.003 |
| ALB | 0.691 | 0.864 |
| A/G | 0.397 | 0.296 |
| DBIL | 0.990 | 0.982 |
| IBIL | 0.003 | *** |
| UREA | 0.078 | 0.034 |
| CR | 0.986 | 0.949 |
| UA | 0.772 | 0.671 |

WBC, White blood cell count. PLT, platelet. RBC, Red blood cell count. HGB, Hemoglobin. HCT, Hematocrit. MCV, Mean corpuscular volume. MCH, Mean corpuscular hemoglobin. MCHC, Mean corpuscular hemoglobin concentration. RDW, Red cell distribution width. RDWSD, Red cell distribution width (standard deviation). N%, Neutrophil percentage. M%, Monocyte percentage. N#, Neutrophil absolute count. M#, Monocyte absolute count. L%, Lymphocyte percentage. E%, Eosinophil percentage. L#, Lymphocyte absolute count. E#, Eosinophil absolute count. B%, Basophil percentage. B#, B%, Basophil absolute count. PA, Prealbumin. TP, Total protein. ALB, Albumin. GLB, Globulin. A/G, Albumin/Globulin ratio. TBIL, Total bilirubin. DBIL, Direct bilirubin. IBIL, Indirect bilirubin. TBA, Total bile acids. ALT, Alanine aminotransferase. AST, Aspartate aminotransferase. ALP, Alkaline phosphatase. GGT, Gamma-glutamyl transferase. LDH, Lactate dehydrogenase. CR, Creatinine. UA, Uric acid. CYSC, Cystatin C. **PWR**, Platelet to White cell Ratio. **NLR**, Neutrophil to Lymphocyte Ratio. **MLR**, Monocyte to Lymphocyte Ratio. **NMLR**, (Neutrophil + Monocyte) to Lymphocyte Ratio. **SIRI**, Systemic Inflammation Response Index. **SII**, Systemic Immune-Inflammation Index. **DNLR**, Derived Neutrophil to Lymphocyte Ratio. **SLR**, AST to ALT Ratio. **UCR,** UREA to Creatinine Ratio. **HGI**, Hemoglobin to Glycerinated Hemoglobin Index. **PNI,** Prognostic Nutrition Index

Supplementary table 4. Obtain variables with significant differences.

| Only RCS | Only cut-off value | RCS and cut-off value |
| --- | --- | --- |
| MCV | PPROM | WBC |
| MCH | Preterm birth | RBC |
| TP | IFD | HGB |
| IBIL | PI | HCT |
| UREA | ICP | M% |
|  | Hypertension | N# |
|  | Preeclampsia | GLB |
|  | Cervical Insufficiency | TBIL |
|  | Chorioamnionitis | SIRI |
|  | Puerperal Infection | DNLR |
|  | Twin Pregnancy | HGI |
|  | Mode of Delivery | PWR |
|  | High-Risk Factors |  |
|  | IVF |  |
|  | Pregnancy |  |
|  | Delivery |  |
|  | Pre-pregnancy Weight |  |
|  | Height |  |
|  | Pre-pregnancy BMI |  |
|  | MCHC |  |
|  | N% |  |
|  | M# |  |
|  | TBA |  |
|  | ALT |  |
|  | AST |  |
|  | ALP |  |
|  | GGT |  |
|  | LDH |  |
|  | CYSC |  |
|  | NLR |  |

MCV, Mean corpuscular volume. MCH, Mean corpuscular hemoglobin. TP, Total protein. IBIL, Indirect bilirubin. PPROM, Preterm premature rupture of membranes. IFD, Intrauterine fetal distress. PI, Placenta invasion. ICP, Intrahepatic cholestasis of pregnancy. IVF, In vitro fertilization. MCHC, Mean corpuscular hemoglobin concentration. N%, Neutrophil percentage. M#, Monocyte absolute count. TBA, Total bile acids. ALT, Alanine aminotransferase. AST, Aspartate aminotransferase. ALP, Alkaline phosphatase. GGT, Gamma-glutamyl transferase. LDH, Lactate dehydrogenase. CYSC, Cystatin C. **NLR**, Neutrophil to Lymphocyte Ratio. WBC, White blood cell count. RBC, Red blood cell count. HGB, Hemoglobin. HCT, Hematocrit. M%, Monocyte percentage. N#, Neutrophil absolute count. GLB, Globulin. TBIL, Total bilirubin. P**WR**, Platelet to White cell Ratio. **SIRI**, Systemic Inflammation Response Index. **DNLR**, Derived Neutrophil to Lymphocyte Ratio. **HGI**, Hemoglobin to Glycerinated Hemoglobin Index.

Supplementary table 5. Multicollinearity diagnosis.

| Variables | Standardized Coefficient | | Significance | Collinearity diagnostics | |
| --- | --- | --- | --- | --- | --- |
|  | Beta | t |  | Tolerance | VIF |
| PPROM | -0.010 | -2.013 | 0.044 | 0.537 | 1.863 |
| Preterm birth | 0.055 | 10.228 | <0.001 | 0.434 | 2.303 |
| IFD | 0.026 | 6.899 | <0.001 | 0.897 | 1.114 |
| PI | 0.009 | 2.511 | 0.012 | 0.936 | 1.069 |
| ICP | 0.011 | 3.140 | 0.002 | 0.956 | 1.046 |
| Hypertension | 0.006 | 1.168 | 0.243 | 0.471 | 2.123 |
| Preeclampsia | -0.003 | -0.491 | 0.624 | 0.468 | 2.136 |
| Cervical Insufficiency | 0.012 | 3.283 | 0.001 | 0.976 | 1.025 |
| Chorioamnionitis | 0.104 | 28.843 | <0.001 | 0.956 | 1.046 |
| Puerperal Infection | 0.010 | 2.802 | 0.005 | 0.994 | 1.006 |
| Twin Pregnancy | 0.015 | 3.777 | <0.001 | 0.826 | 1.211 |
| Mode of Delivery | -0.020 | -4.885 | <0.001 | 0.738 | 1.354 |
| WBC | -0.019 | -0.624 | 0.532 | 0.013 | 74.719 |
| RBC | 0.024 | 3.348 | 0.001 | 0.246 | 4.058 |
| HGB | 0.018 | 1.276 | 0.202 | 0.062 | 16.021 |
| HCT | -0.034 | -2.175 | 0.030 | 0.051 | 19.668 |
| M_Per | -0.002 | -0.247 | 0.805 | 0.283 | 3.528 |
| N_Jue | 0.009 | 0.248 | 0.804 | 0.010 | 100.846 |
| GLB | 0.004 | 1.076 | 0.282 | 0.942 | 1.061 |
| SIRI | 0.019 | 2.650 | 0.008 | 0.231 | 4.327 |
| DNLR | -0.007 | -0.870 | 0.384 | 0.188 | 5.312 |
| HGI | 0.010 | 1.710 | 0.087 | 0.353 | 2.829 |
| PWR | 0.006 | 1.102 | 0.270 | 0.484 | 2.065 |
| TBIL | -0.003 | -0.867 | 0.386 | 0.952 | 1.051 |
| High-Risk Factors | 0.005 | 1.182 | 0.237 | 0.718 | 1.392 |
| IVF | -0.002 | -0.426 | 0.670 | 0.715 | 1.398 |
| Pregnancy | -0.003 | -0.422 | 0.673 | 0.197 | 5.069 |
| Delivery | -0.005 | -0.950 | 0.342 | 0.442 | 2.262 |

WBC, White blood cell count. PLT, platelet. RBC, Red blood cell count. HGB, Hemoglobin. HCT, Hematocrit. MCV, Mean corpuscular volume. MCH, Mean corpuscular hemoglobin. MCHC, Mean corpuscular hemoglobin concentration. RDW, Red cell distribution width. RDWSD, Red cell distribution width (standard deviation). N%, Neutrophil percentage. M%, Monocyte percentage. N#, Neutrophil absolute count. M#, Monocyte absolute count. L%, Lymphocyte percentage. E%, Eosinophil percentage. L#, Lymphocyte absolute count. E#, Eosinophil absolute count. B%, Basophil percentage. B#, B%, Basophil absolute count. PA, Prealbumin. TP, Total protein. ALB, Albumin. GLB, Globulin. A/G, Albumin/Globulin ratio. TBIL, Total bilirubin. DBIL, Direct bilirubin. IBIL, Indirect bilirubin. TBA, Total bile acids. ALT, Alanine aminotransferase. AST, Aspartate aminotransferase. ALP, Alkaline phosphatase. GGT, Gamma-glutamyl transferase. LDH, Lactate dehydrogenase. CR, Creatinine. UA, Uric acid. CYSC, Cystatin C. **PWR**, Platelet to White cell Ratio. **NLR**, Neutrophil to Lymphocyte Ratio. **MLR**, Monocyte to Lymphocyte Ratio. **NMLR**, (Neutrophil + Monocyte) to Lymphocyte Ratio. **SIRI**, Systemic Inflammation Response Index . **SII**, Systemic Immune-Inflammation Index. **DNLR**, Derived Neutrophil to Lymphocyte Ratio. **SLR**, AST to ALT Ratio. **UCR,** UREA to Creatinine Ratio. **HGI**, Hemoglobin to Glycerinated Hemoglobin Index. **PNI,** Prognostic Nutrition Index. VIF, Variance Inflation Factor. PPROM, Preterm premature rupture of membranes. IFD, Intrauterine fetal distress. PI, Placenta invasion. ICP, Intrahepatic cholestasis of pregnancy. IVF, In vitro fertilization.
